# The Effects of Vitamin D_3_ Supplementation in Late Gestation on Lactation Performance, Constipation and Intestinal Microbiota of Sows

**DOI:** 10.3390/ani16172690

**Published:** 2026-08-28

**Authors:** Yujiao Chen, Tanghong Wei, Liudan Liu, Ping Lin, Zhenxing Wang, Zhijian Tan, Xiangfang Zeng, Chengquan Tan, Haiqing Sun, Ming Deng

**Affiliations:** 1 State Key Laboratory of Swine and Poultry Breeding Industry, College of Animal Science, South China Agricultural University, Guangzhou 510642, China; 2 College of Animal Science & Technology, Huazhong Agricultural University, Wuhan 430070, China; 3 Dekang Food and Agriculture Group Co., Ltd., Chengdu 610200, China; 4 Foshan Standard Bio-Tech Co., Ltd., Foshan 528138, China; 5 State Key Laboratory of Animal Nutrition and Feeding, Ministry of Agriculture and Rural Affairs Feed Industry Center, China Agricultural University, Beijing 430070, China; 6 Guangxi Yangxiang Group Co., Ltd., Guigang 537100, China

**Keywords:** constipation, gut microbiota, peripartum health, sow, vitamin D_3_, weaning survival rate

## Abstract

This study was conducted to systematically evaluate the effects of dietary vitamin D_3_ supplementation during late gestation on constipation, gut microbiota, and lactation performance in sows. A total of 42 healthy sows with similar parity and gestational status were randomly assigned to two groups on gestation day 75 (G 75): CON (basal diet), VD_3_ (2340 IU/kg was supplemented daily from G 75 to G 90). Analysis of constipation-related indicators showed that the incidence of constipation before (*p* < 0.05) and after farrowing (*p* < 0.01) was significantly lower in the vitamin D_3_ supplementation group, and constipation cases in this group were predominantly classified as mild (*p* < 0.05). In addition, fecal moisture content was significantly higher in the vitamin D_3_ supplementation group on G 109 (*p* < 0.05). Inflammation-related marker analysis showed that, compared with the CON group, sows in the VD_3_ group had significantly lower fecal calprotectin concentrations on G 109 (*p* < 0.05). Further Spearman correlation analysis showed that fecal calprotectin concentration was significantly negatively correlated with piglet weaning survival rate (*p* < 0.01). Gut microbiota analysis showed that vitamin D_3_ supplementation significantly increased the relative abundance of *Clostridium* and altered gut microbial composition and predicted KEGG functional profiles in sows. Although the difference did not reach statistical significance, piglets in the VD_3_ group showed a tendency toward a higher weaning survival rate (*p* = 0.08). Overall, these findings indicate that late-gestation dietary vitamin D_3_ supplementation was associated with a lower incidence of peripartum constipation and alterations in gut microbial community composition and predicted functional profiles in sows. Its potential effects on piglet weaning outcomes require further investigation in studies with larger sample sizes.

## Simple Summary

Dietary vitamin D_3_ supplementation during late gestation modulated gut microbiota composition, and reduced peripartum constipation-related indicators in sows. These changes may be associated with the weaning survival rate of piglets. These findings provide supportive evidence for supplementing sows diets with vitamin D_3_ during late gestation to support peripartum intestinal health and lactation-related outcomes.

## 1. Introduction

In large-scale pig production, the reproductive performance of sows depends on two critical periods: gestation and lactation [1]. During the transition from pregnancy to lactation, sows undergo profound physiological, metabolic, and immune remodeling to meet the nutritional and energy demands of fetal development, parturition, and lactogenesis [2]. Restricted feeding strategy is widely adopted in commercial pig farms to regulate sow body condition and sustain their normal reproductive performance [3,4,5]. However, the combined effects of restricted feeding-induced insufficient gastrointestinal digesta [6], mechanical compression of the gastrointestinal tract by the enlarged uterus in late gestation [7], and the direct inhibitory effect of high progesterone concentrations on gastrointestinal smooth muscle motility collectively lead to a marked reduction in intestinal motility [8,9]. This prolongs colonic fecal retention time, causing excessive water reabsorption and ultimately inducing physiological constipation in sows. In sows, constipation during late gestation is commonly associated with reduced intestinal motility and prolonged fecal retention [10,11] and may contribute to gut microbial dysbiosis and alterations in intestinal mucosal immune homeostasis [12]. Peripartum constipation may also interfere with the normal farrowing process and increase the risk of dystocia [13,14,15]. Furthermore, reductions in feed intake and disturbances in nutrient metabolism associated with constipation may adversely affect lactation performance and, consequently, impair piglet growth and survival during the suckling period [16]. Therefore, maintaining sow metabolic health and alleviating constipation during late gestation and lactation are crucial for ensuring sow reproductive performance and promoting the healthy growth of offspring piglets.

Vitamin D_3_ is an inactive form of vitamin D that is converted to 25-hydroxyvitamin D_3_ (25OHD_3_) in the liver and further hydroxylated in the kidneys to produce its active form, 1, 25-dihydroxyvitamin D_3_ [1, 25(OH)_2_D_3_] [17]. Vitamin D_3_ is primarily acquired via two pathways: first, through dietary ingestion of exogenous vitamin D_3_; second, through the photochemical synthesis of endogenous vitamin D_3_ in the skin upon ultraviolet radiation exposure, a reaction where epidermal 7-dehydrocholesterol is converted to this active precursor [18]. However, under modern intensive swine production conditions, sow herds are typically housed indoors in high-density stalls with restricted movement and negligible natural sunlight exposure. Therefore, dietary supplementation is essential to maintain physiologically normal levels of vitamin D_3_ in sows. As vitamin D_3_ is a fat-soluble vitamin, prolonged intake or exposure to excessively high dietary levels may result in excessive accumulation of vitamin D metabolites and induce toxic effects, including hypercalcemia and soft-tissue mineralization [19,20], through disruption of calcium and phosphorus homeostasis. Therefore, both the dietary inclusion level and supplementation duration of vitamin D_3_ should be carefully controlled. When its dietary inclusion level and supplementation duration are appropriately controlled, vitamin D_3_, in addition to its role in calcium and phosphorus homeostasis, may contribute to the regulation of intestinal barrier function and mucosal immunity through the vitamin D receptor (VDR), thereby potentially influencing gut microbial composition [21,22]. Animal studies have shown that vitamin D_3_ supplementation can alter gut microbial composition [23]; in mice with high-fat diet-induced obesity, these changes were accompanied by improved intestinal barrier function and reduced inflammatory markers [24].

Existing studies have mainly focused on the regulatory effects of vitamin D_3_ and its metabolites on reproductive performance, bone metabolism, and postpartum health in gestating sows, as well as growth performance in weaned piglets [25,26,27,28,29]. However, investigations into the effects of vitamin D_3_ on gut microbiota composition, constipation, and immune function in sows remain limited. However, clear evidence regarding the effects of vitamin D_3_ on the gut microbiota of peripartum sows and constipation-related health indicators remains lacking.

In this study, vitamin D_3_ supplementation was provided to sows from G 75 to G 90 to investigate its effects on gut microbiota, late-gestational inflammatory responses, and constipation before and after farrowing. By integrating sow reproductive performance with piglet growth outcomes, this study further aimed to elucidate the potential of vitamin D_3_ supplementation to modulate intestinal health and periparturient physiological status in late-gestation sows.

## 2. Materials and Methods

### 2.1. Ethics Statement, Experiment Design, and Management

All animal experiments in this study were approved by the Animal Care and Use Committee of the Subtropical Agriculture Research Institute, Chinese Academy of Sciences, and conducted in accordance with the Guidelines for the Care and Use of Laboratory Animals at South China Agricultural University (2026G001).

This study was conducted at a breeding farm in Guigang, China (affiliated with Guangxi Yangxiang Agriculture and Animal Husbandry Co., Ltd.). The experimental facility was not a multi-story pig building. The barn was equipped with negative-pressure ventilation and a fan-pad evaporative cooling system to regulate the indoor environment. Temperature and relative humidity were routinely monitored and maintained within the farm’s standard management range for gestating and periparturient sows. A total of 42 healthy pregnant sows of Danish Landrace and Yorkshire breeds, with parities ranging from 2 to 4, were randomly assigned on G 75 to either CON group or VD_3_ group, with 21 sows per group. Each sow was considered an independent experimental unit. Of the 42 sows, 40 completed the study and were included in the final analysis, with 20 sows in each group. Two sows were excluded: one sow in the CON group exhibited abnormal feed intake during lactation, and one sow in the VD_3_ group died during gestation. The experimental period lasted from G 75 to lactational day 21 (L 21). From G 75 until transfer to the farrowing room, sows were housed individually in gestation stalls. During the gestation, each sow was subjected to restricted feeding management (daily feed allowance of 2.0 kg from G 75 to G 90, and 2.8 kg from G 91 until the day before farrowing), with feeding administered once daily at 7:00 AM. Sows were moved to the farrowing room 3–5 days before the expected farrowing and housed individually in raised farrowing crates. These stalls consist of components such as a sow positioning frame, piglet pen, slatted floor, heat lamp, and feed trough. During the lactation, sows were fed twice daily at 7:00 AM and 3:30 PM with a lactation diet. Following farrowing, a restricted feeding regimen was implemented for the first three days, after which feed intake was gradually increased until transitioning to ad libitum feeding. Heat lamps were installed above the farrowing crates to maintain piglet temperature. Sows were provided with clean drinking water throughout the trial. All animal management procedures were conducted in accordance with the approved animal care and use protocol.

The vitamin D_3_ used in this study was provided by Foshan Standard Bio-tech Co., Ltd. (Foshan, China), with a biological activity of 670,000 IU/kg. During the trial period, stepwise feeding operations were conducted from G 75 to G 90: in addition to the basal diet, 4 g of a vitamin D_3_ supplement per sow per day was individually weighed and administered separately to each sow in the VD_3_ group. The final vitamin D_3_ concentration in the diet of the VD_3_ group was 2340 IU/kg. All experimental diets were formulated based on the National Research Council’s nutritional guidelines [30]. Their composition and nutritional levels are detailed in Supplementary Table S1. Chemical concentration levels were calculated based on feed ingredient data provided by the National Research Council (2012) [30]. The crude protein, crude fat, crude ash, and crude fiber contents in the experimental diets were determined according to the Chinese national standard GB/T 18868—2024 [31]. Each sow served as an independent experimental unit.

### 2.2. Measurements of Reproductive Performance

Quantitative assessments of backfat thickness, chest circumference, and body length were conducted on all sows at G 75, G 110, and day of farrowing to calculate weight changes. In this study, sow body weight was estimated using a tape-measure-based body measurement method, as previously described for pigs and sows with minor modification [32,33].

During farrowing, trained farrowing attendants continuously monitored each sow and recorded the birth times of the first and last piglets to the nearest minute. Farrowing duration was defined as the total time from the birth of the first piglet to the birth of the last piglet and was expressed in minutes. Farrowing interval was calculated as farrowing duration divided by the total number of piglets born. Sow reproductive performance was systematically recorded post-farrowing, including total litter weight, total number of piglets born, number of live-born piglets, number of stillborn piglets, number of mummified fetuses and number of intrauterine growth restriction (IUGR) piglets. Live-born rate was calculated as the proportion of live piglets to the total farrowed piglets. Stillbirth rate was calculated as the proportion of stillborn piglets to total farrowed piglets. The uniformity of newborn piglets was determined by the coefficient of variation (CV), calculated as the standard deviation of birth weight within a litter divided by the average birth weight of piglets within that litter and multiplied by 100. IUGR was defined in piglets by the diagnostic criterion that their birth weight is two standard deviations below the mean value (mean − 2 SD).

After farrowing, piglets were cross-fostered only among sows within the same dietary treatment, with no cross-fostering between different treatment groups. Cross-fostering was completed within 48 h after farrowing to standardize litter size to approximately 12 piglets per sow. At cross-fostering and weaning, all live piglets in each litter were weighed using an electronic scale, and the number of live piglets was recorded. Litter weight was calculated as the sum of individual piglet weights. Piglet average daily gain (ADG) and survival rate during lactation were calculated based on litter weight and live piglet number. Sow average daily feed intake (ADFI) during lactation was also recorded. All piglets in this study were weaned at 21 days of age.

### 2.3. Determination of Fecal Scoring and Evaluation of Constipation Severity and Rate During Pregnancy

From 5 days before farrowing to 5 days after farrowing, sow fecal scores were assessed daily at 7:00 AM. Fecal scores were evaluated post-defecation using a visual qualitative method. Using a scoring scale from earlier studies, scores ranged from 0 to 5:0 = No feces present 1 = Dry and granular 2 = Texture between dry and normal 3 = Normal, soft but firm and well-formed 4 = Between normal and moist, still forming but not firm 5 = Very moist, unformed, and liquid feces [34], scoring was based on observing signs of consistency in feces from individual pigs and within pens. Constipation severity was classified according to consecutive days without bowel movements: mild (no bowel movement for 2 consecutive days), severe (3–4 consecutive days), and extremely severe (≥5 consecutive days) [11]. Additionally, this trial used a fecal score < 3 as the criterion for determining constipation in sows, thereby calculating the constipation rate for both group [35].

### 2.4. Fecal Samples and Blood Samples of Sows

At G 109, 10 clinically healthy sows were randomly selected from each group, and fresh fecal samples were collected via rectal massage. This time point was selected to evaluate the maternal intestinal status after the completion of vitamin D_3_ supplementation and before farrowing, thereby providing information on the potential carry-over effects of late-gestation vitamin D_3_ supplementation. Immediately after collection, the surface layer of feces was removed to minimize environmental contamination. The inner layer of feces was then divided into sterile cryogenic tubes and rapidly stored at −20 °C for subsequent analysis. In this experiment, 10 clinically healthy and representative sows were randomly selected from each group for fecal sample collection.

During farrowing, 10 clinically healthy and representative sows were randomly selected from each group for blood sample collection, and blood samples were collected via the marginal ear vein. After standing for 30 min, the samples were centrifuged (3000 rpm, 4 °C, 10 min), aliquoted, and stored at −20 °C for subsequent testing and analysis.

### 2.5. Measurement of Fecal Moisture Content, Fecal Calprotectin, and Serum Tumor Necrosis Factor-α (TNF-α)

Serum samples and fecal samples from sows were used to measure the levels of TNF-α and calprotectin with commercial kits (Jiangsu Meimian Industrial Co., Ltd., Yancheng, China).

The collected fecal samples were thawed at 4 °C and then air-dried at 65 °C for 48 h to determine free water content. Subsequently, the samples were equilibrated at 105 °C for 7–8 h until reaching a constant weight to determine total moisture content [36,37].

### 2.6. 16S rRNA Gene Amplicon Sequencing

PCR amplification of the bacterial 16S rRNA genes V3–V4 region was performed using the forward primer 338F (5′-ACTCCTACGGGAGGCAGCA-3′) and the reverse primer 806R (5′-GGACTACHVGGGTWTCTAAT-3′). Sample-specific 7 bp barcodes were incorporated into the primers for multiplex sequencing. The PCR components contained 5 μL of buffer (5×), 0.25 μL of Fast pfu DNA Polymerase (5 U/μL), 2 μL (2.5 mM) of dNTPs, 1 μL (10 μM) of each Forward and Reverse primer, 1 μL of DNA Template, and 14.75 μL of ddH_2_O. Thermal cycling consisted of initial denaturation at 98 °C for 5 min, followed by 25 cycles consisting of denaturation at 98 °C for 30 s, annealing at 53 °C for 30 s, and extension at 72 °C for 45 s, with a final extension of 5 min at 72 °C. PCR amplicons were purified with Vazyme VAHTSTM DNA Clean Beads (Vazyme, Nanjing, China) and quantified using the Quant-iT PicoGreen dsDNA Assay Kit (Invitrogen, Carlsbad, CA, USA). After the individual quantification step, amplicons were pooled in equal amounts, and pair-end 2 × 250 bp sequencing was performed using the Illlumina NovaSeq platform with NovaSeq 6000 SP Reagent Kit (500 cycles) at Shanghai Personal Biotechnology Co., Ltd. (Shanghai, China).

### 2.7. Statistical Analysis

#### 2.7.1. Statistical Analysis of Phenotypic and Physiological Data

Data analysis was performed using SPSS 26.0. All data were first tested for normality using the Kolmogorov–Smirnov test and for homogeneity of variance using Levene’s test. When both the normality and homogeneity of variance assumptions were satisfied, between-group comparisons were performed using an independent-samples *t*-test. When either assumption was violated, the Mann–Whitney U test was used, as indicated in the corresponding table footnotes. For categorical variables or proportional data, the chi-square test was used according to the data characteristics, as indicated in the corresponding table footnotes.

#### 2.7.2. Statistical Analysis of 16S rRNA Gene Sequencing Data

For fecal microbiota analysis based on 16S rRNA gene sequencing, bioinformatic analysis was performed using QIIME2 (version 2019.4), with appropriate modifications and optimization according to the official tutorials. Raw sequence data were first demultiplexed using the demux plugin, and primer sequences were removed using the cutadapt plugin. Subsequently, the DADA2 plugin was used for quality filtering, denoising, paired-end read merging, and chimera removal, generating amplicon sequence variants (ASVs) and the corresponding abundance table. Taxonomic assignment of representative ASV sequences was performed using the Greengenes2 database (version 2022.10) to obtain taxonomic information for each ASV. ASVs with an abundance lower than 0.001% of the total sequencing depth across all samples were removed, and the abundance matrix after rare ASV removal was used for subsequent analyses.

For alpha diversity analysis, random subsampling was first performed at different sequencing depths based on the ASV abundance matrix, and rarefaction curves were generated to evaluate whether the sequencing depth was sufficient to reflect microbial diversity within each sample. Subsequently, the ASV abundance matrix was rarefied to 95% of the sequencing depth of the sample with the lowest number of sequences across all samples to reduce the influence of differences in sequencing depth on diversity comparisons. Based on the rarefied ASV abundance matrix, Chao1, Shannon, and Simpson indices were calculated for each sample using QIIME2 to compare microbial richness and evenness among samples. Beta diversity analysis was performed using QIIME2 and R (version 4.3.3). Differences in microbial community structure among samples were assessed based on Bray–Curtis dissimilarity and visualized using principal coordinates analysis (PCoA) with the R packages ape (version 5.8) and vegan (version 2.6-6.1). Statistical differences in beta diversity between groups were evaluated using Adonis analysis, a permutational multivariate analysis of variance, based on the Bray–Curtis distance matrix.

Differentially abundant bacterial taxa at the genus level were identified using linear discriminant analysis effect size (LEfSe). LEfSe analysis was performed using the Python (version 3.9) LEfSe package with a one-against-all comparison strategy, the Wilcoxon test enabled, and an LDA score threshold > 3 for identifying discriminative taxa. Other between-group comparisons of microbiota-related variables were performed using the Mann–Whitney U test.

Microbial metabolic functions were predicted using Phylogenetic Investigation of Communities by Reconstruction of Unobserved States 2 (PICRUSt2) (version 2.2.2-b) with its embedded Kyoto Encyclopedia of Genes and Genomes (KEGG) pathway annotation workflow. Predicted functional pathways were compared between groups based on the resulting pathway abundance profiles.

Except for the visualizations specified above, all other figures were generated using GraphPad Prism 8. Results are presented as the mean ± standard error of the mean (SEM). Statistical significance was indicated as follows: *, *p* < 0.05; **, *p* < 0.01; ***, *p* < 0.001; trend, 0.05 ≤ *p* < 0.10; ns, *p* ≥ 0.1.

## 3. Results

### 3.1. Body Weight, Backfat Thickness and Lactation Feed Intake of Sows

As shown in Table 1, supplementing vitamin D_3_ in the diets of sows in late gestation had no significant effect on backfat thickness, body weight, or ADFI during the lactation period (*p* > 0.05).

### 3.2. Reproductive Performance of Sows

As shown in Table 2, no significant differences were observed in reproductive performance between the two groups (*p* > 0.05). Although farrowing duration was approximately 30 min shorter and the number of IUGR piglets was numerically lower in the VD_3_ group than in the CON group, these differences did not reach statistical significance.

### 3.3. Piglet Growth Performance

As shown in Table 3, there were no significant differences in the relevant parameters between the CON and VD_3_ groups at cross-fostering (*p* > 0.05). At weaning on L 21, the number of piglets, average piglet weight, litter weight, and ADG did not differ significantly between the two groups (*p* > 0.05). However, compared with the CON group, the weaning survival rate of piglets in the VD_3_ group tended to increase (*p* = 0.084).

### 3.4. Fecal and Constipation Indices of Sows

**Figure 1:**
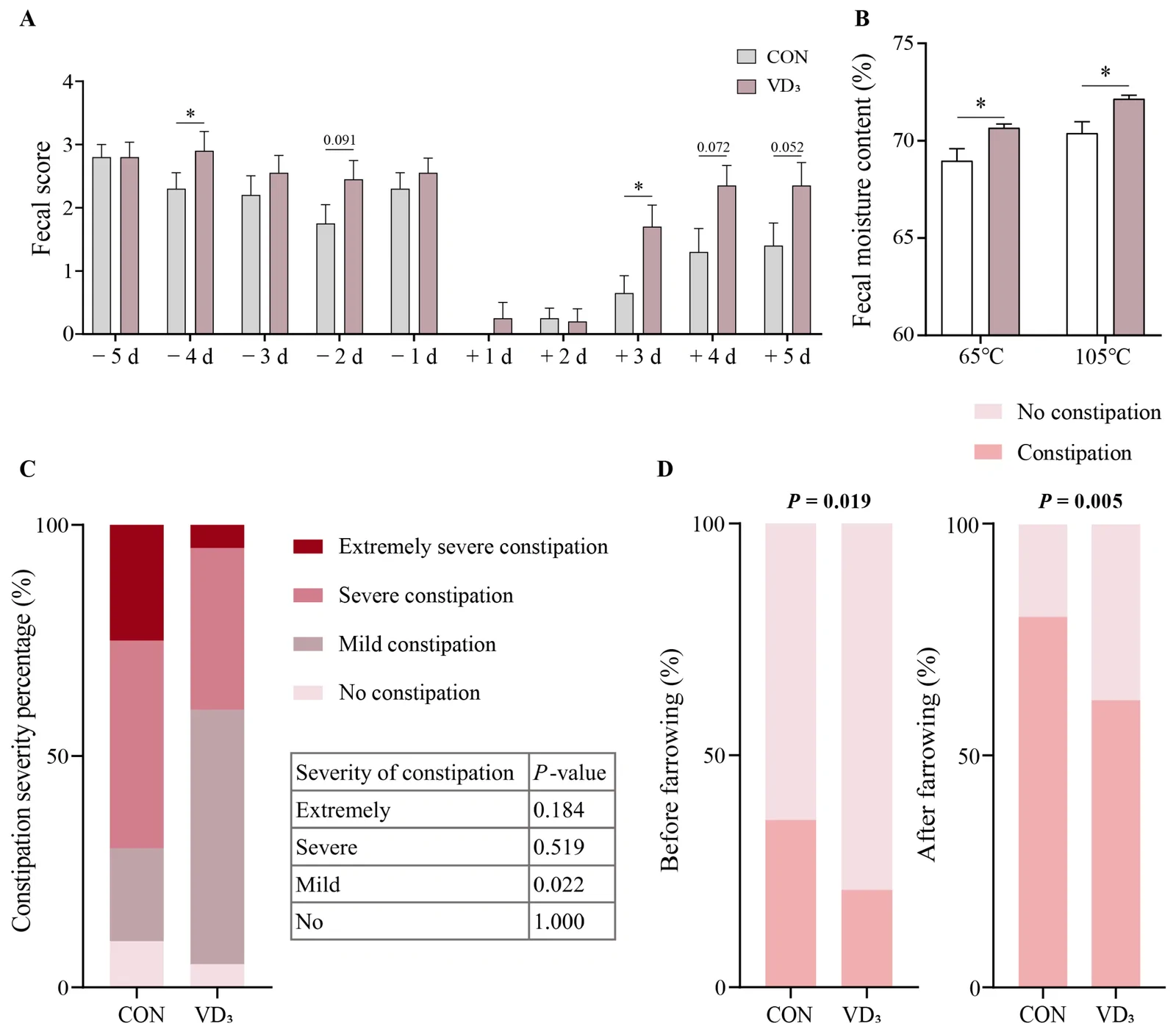
Effects of dietary vitamin D_3_ supplementation on constipation in sows. (**A**) Fecal score of sows within 5 days before and after farrowing (*n* = 20). (**B**) Free water content in sow feces after natural air-drying at 65 °C and total water content after drying to constant weight at 105 °C at gestational day 109. (*n* = 10, 9). (**C**) Distribution of constipation severity in sows (*n* = 20). (**D**) Constipation rates of sows before and after farrowing (*n* = 20). Data are expressed as mean ± SEM. * *p* < 0.05; values without marks indicate no significance (*p* > 0.05). The negative sign denotes “before farrowing”; the plus sign denotes “after farrowing”. CON = Control group; VD_3_ = Vitamin D_3_ supplementation group, with a dietary vitamin D_3_ activity of 2340 IU/kg from G 75 to G 90.

As shown in Figure 1A, dietary vitamin D_3_ supplementation significantly increased sow fecal scores 4 days before farrowing and 3 days after farrowing (*p* < 0.05), and sow fecal scores 5 days after farrowing showed an increasing trend (*p* = 0.052). In addition, fecal moisture content measured at 65 °C and 105 °C was significantly higher in the VD_3_ group than in the CON group, increasing by 1.69 and 1.77 percentage points, respectively. (Figure 1B). The distribution of constipation severity showed that, compared with the CON group, the proportion of sows with mild constipation was significantly increased in the VD_3_ group (*p* < 0.05); whereas, the proportions of sows with severe and extremely severe constipation were numerically decreased but did not reach statistical significance (Figure 1C). Further analysis showed that the prepartum constipation rate was significantly lower in the VD_3_ group than in the CON group (*p* < 0.05), and the postpartum constipation rate was also significantly lower in the VD_3_ group (*p* < 0.01) (Figure 1D).

### 3.5. Chemical Analysis of Maternal Inflammation Factor in Sows

**Figure 2:**
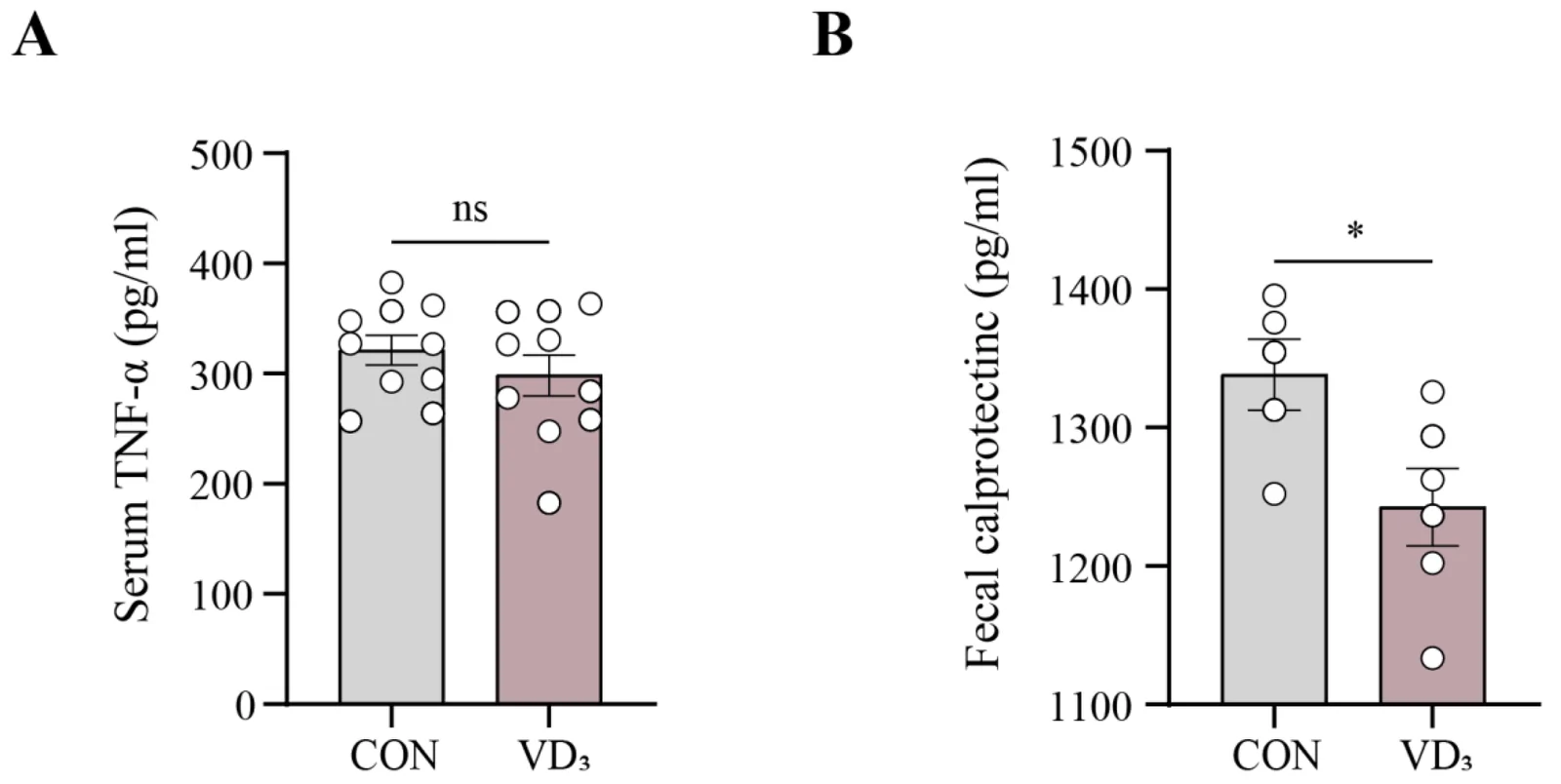
Effects of dietary vitamin D_3_ supplementation on the inflammatory response of late-gestation sows. (**A**) TNF-α (Tumor necrosis factor α) concentrations in sow serum samples during farrowing (*n* = 10). (**B**) Fecal calprotectin concentrations in sows at gestational day 109 (*n* = 5, 6). Data are expressed as mean ± SEM. * *p* < 0.05; ns, not significant (*p* > 0.05). CON = Control group; VD_3_ = Vitamin D_3_ supplementation group, with a dietary vitamin D_3_ activity of 2340 IU/kg from G 75 to G 90.

No significant difference in serum TNF-α concentration at farrowing was observed between the CON and VD_3_ groups (Figure 2A). In contrast, fecal calprotectin concentration in sows was significantly lower in the VD_3_ group than in the CON group at G 109 (Figure 2B).

### 3.6. Fecal Microbiota Based on 16S rRNA Gene Sequencing

To investigate whether dietary vitamin D_3_ supplementation during late gestation affected sow gut microbiota, fecal microbiota profiles at G 109 were compared between the CON and VD_3_ group. Following quality control of 16S rRNA gene sequencing data from fecal samples, a total of 1,102,987 valid tags were obtained from 20 fecal samples, with an average of 55,149 valid tags per sample. The coverage rate of all samples reached 99.99%, demonstrating that the sequencing data sufficiently captured the community distribution of fecal bacteria.

**Figure 3:**
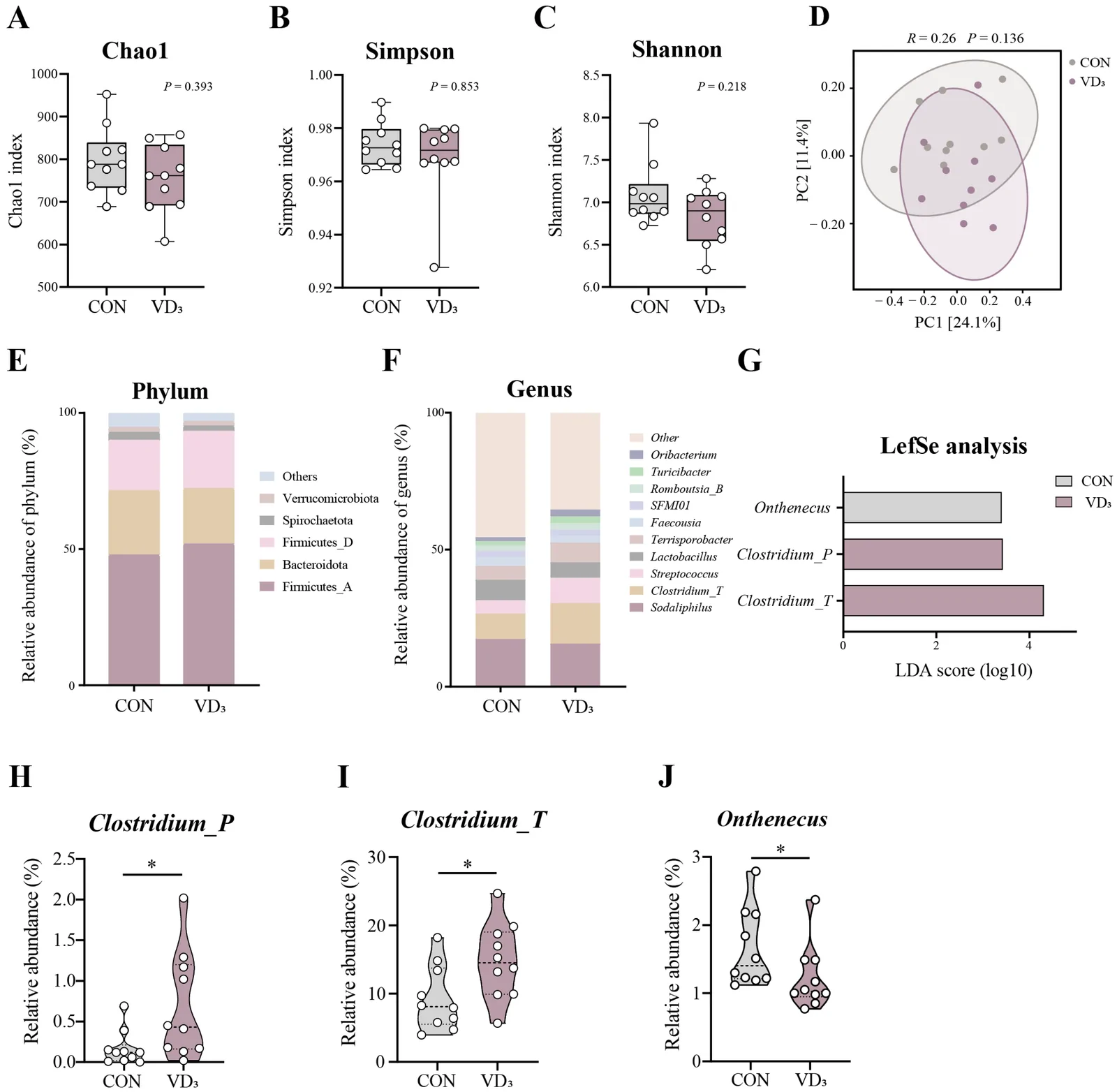
Effects of dietary vitamin D_3_ supplementation on the intestinal microbiota of late-gestation sows. Alpha diversity indices: Chao1 (**A**), Simpson (**B**), and Shannon (**C**). (**D**) Principal coordinate analysis of gut microbiota composition. (**E**) Relative abundance of the top 5 bacterial phyla in fecal microbiota. (**F**) Relative abundance of the top 10 bacterial genera in fecal microbiota. (**G**) LEfSe analysis identifying genus-level microbial taxa with high biological contribution (LDA scores > 3.0). (**H**–**J**) Relative abundances of LEfSe-derived differential genera between the CON and VD_3_ groups. * *p* < 0.05, *n* = 10; all difference analyses employed the Mann–Whitney U test. CON = Control group; VD_3_ = Vitamin D_3_ supplementation group, with a dietary vitamin D_3_ activity of 2340 IU/kg from G 75 to G 90.

Alpha diversity analysis showed that Chao1, Simpson, and Shannon indices did not differ significantly between the two groups (*p* > 0.05) (Figure 3A–C). Beta diversity was assessed using Bray–Curtis distance and visualized by PCoA. As shown in Figure 3D, the CON and VD_3_ groups showed partial separation in fecal microbial community structure. However, Adonis analysis based on the Bray–Curtis dissimilarity matrix indicated that the difference between the two groups was not statistically significant (*p* > 0.05).

Subsequently, the effects of dietary vitamin D_3_ supplementation during late gestation on the gut microbiota composition were investigated. At the phylum level, the dominant phyla in the sow gut microbiota were Firmicutes and Bacteroidetes, which together accounted for more than 90% of the total relative abundance (Figure 3E). At the genus level, the top 10 genera by relative abundance were *Sodaliphilus*, *Clostridium_T*, *Streptococcus*, *Lactobacillus*, *Terrisporobacter*, *Faecousia*, *SFMI01*, *Romboutsia_B*, *Turicibacter*, and *Oribacterium*. The total relative abundance of these genera was approximately 54% in the CON group and 64% in the VD_3_ group (Figure 3F).

LEfSe analysis was performed to identify differentially abundant genera between the CON and VD_3_ groups. As shown in Figure 3G, *Clostridium_T* and *Clostridium_P* were enriched in the VD_3_ group; whereas, *Onthenecus* was enriched in the CON group, based on an LDA score threshold > 3. These genera were identified as differentially abundant genera by LEfSe analysis. To further validate the above findings, we performed separate between-group comparisons of the relative abundance of the three genera. The results showed that the relative abundances of all three genera differed significantly between the CON and VD_3_ groups (*p* < 0.05) (Figure 3H–J).

### 3.7. Functional Prediction and Analysis of Pathways Based on the KEGG Database

**Figure 4:**
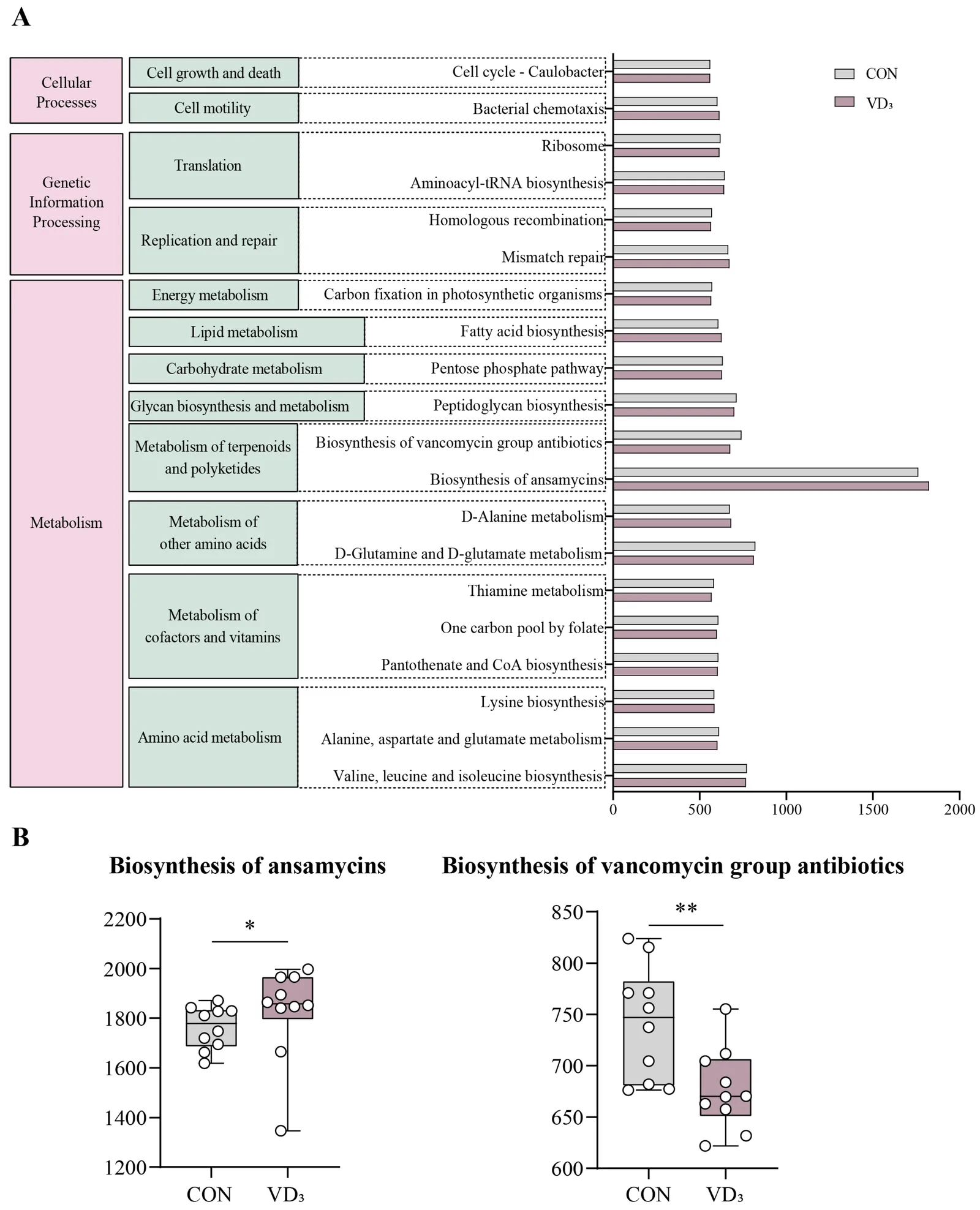
Effects of dietary vitamin D_3_ supplementation on KEGG functional annotation of intestinal microbiota in late-gestation sows. (**A**) Functional predictions based on the KEGG database (Top 20 pathways ranked by abundance). (**B**) Comparison of the top 20 most abundant predicted KEGG pathways between the CON and VD_3_ groups based on PICRUSt2 analysis. * *p* < 0.05, ** *p* < 0.01, *n* = 10; using the Mann–Whitney U test. CON = Control group; VD_3_ = Vitamin D_3_ supplementation group, with a dietary vitamin D_3_ activity of 2340 IU/kg from G 75 to G 90.

To further explore potential functional differences in the fecal microbiota, microbial functional profiles were predicted using PICRUSt2 based on 16S rRNA gene sequencing data. First, the top 20 most abundant metabolic pathways in both sample groups were categorized according to KEGG Level 3 classification. Combined with their corresponding Level 2 and Level 1 information, the abundance of each pathway was presented in a bar chart (Figure 4A). The results indicate that the dominant pathways are primarily concentrated in Metabolism/Metabolism of Terpenoids and Polyketides, with the highest relative abundance observed in the biosynthesis of ansamycins pathway. The top 20 most abundant predicted KEGG pathways were further compared between the CON and VD_3_ groups. As shown in Figure 4B, before multiple testing correction, the predicted vancomycin group antibiotics biosynthesis pathway was significantly lower in the VD_3_ group than in the CON group (*p* < 0.01); whereas, the predicted ansamycin pathway was significantly higher in the VD_3_ group (*p* < 0.05). However, after Benjamini–Hochberg FDR correction, these pathway differences did not remain statistically significant (FDR > 0.05). Therefore, these predicted functional differences should be interpreted as exploratory findings rather than experimentally confirmed metabolic changes.

### 3.8. Spearman Correlation Analysis Among Fecal Traits, Microbial Taxa, Predicted Metabolic Pathways, and Reproductive Performance Indicators

**Figure 5:**
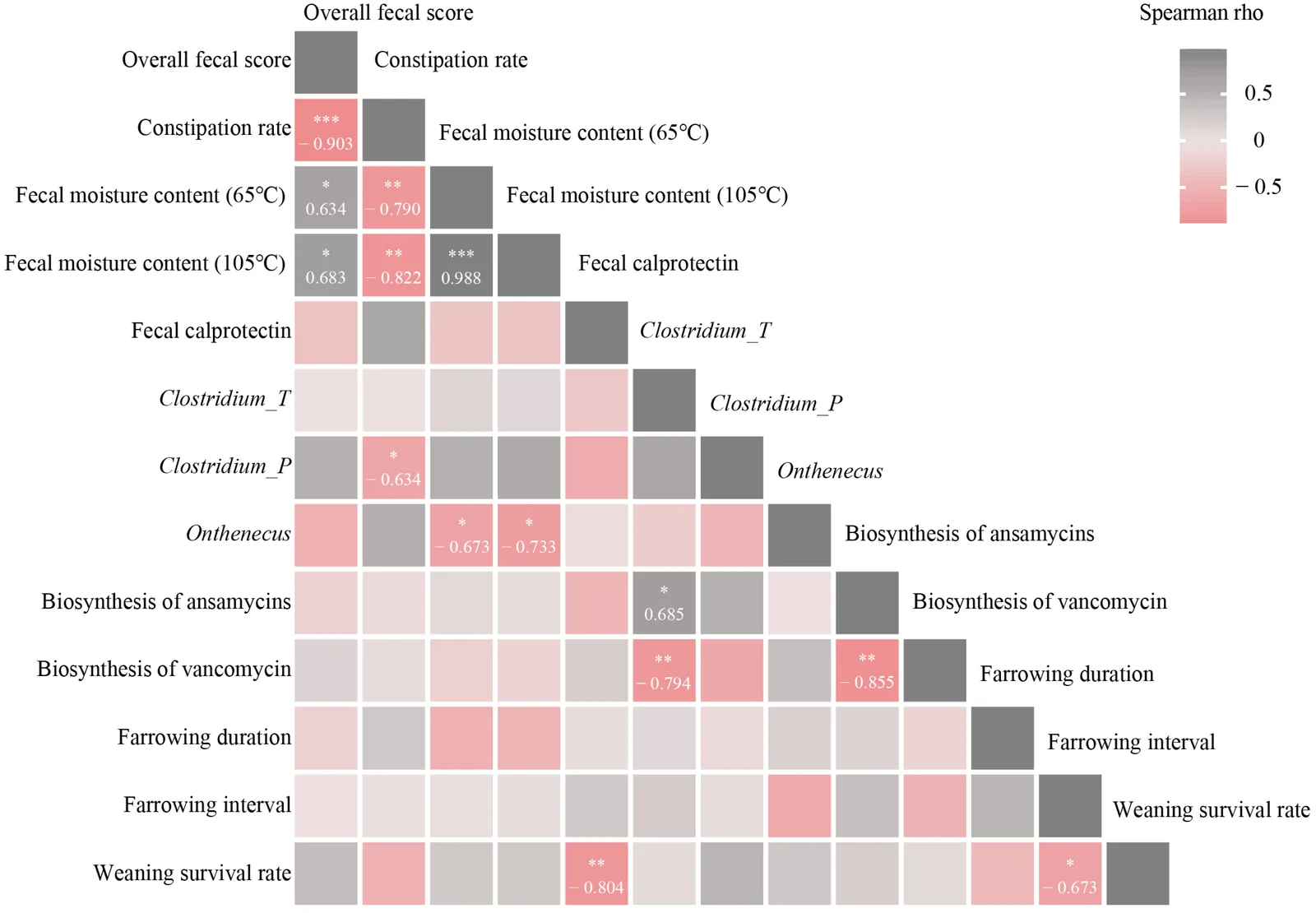
Spearman’s correlation analysis of fecal traits, microbial taxa, metabolic pathways and reproductive performance indicators. Overall fecal score was defined as the mean of sow fecal scores within 5 days before and after farrowing (10 days total). * *p* < 0.05; ** *p* < 0.01; *** *p* < 0.001; *n* = 10. CON = Control group; VD_3_ = Vitamin D_3_ supplementation group, with a dietary vitamin D_3_ activity of 2340 IU/kg from G 75 to G 90.

To explore potential associations among fecal traits, microbial taxa, predicted metabolic pathways, and reproductive performance indicators, Spearman correlation analysis was performed. As shown in Figure 5, several significant correlations were observed among these variables. Specifically, fecal moisture content measured at 65 °C and 105 °C was positively correlated with overall fecal score and negatively correlated with constipation rate (*p* < 0.05); overall fecal score was also negatively correlated with constipation rate (*p* < 0.001). Among microbial taxa, *Clostridium_P* was negatively correlated with constipation rate (*p* < 0.05); whereas, *Onthenecus* was negatively correlated with fecal moisture content measured at both 65 °C and 105 °C (*p* < 0.05). In addition, the predicted metabolic pathways Biosynthesis of ansamycins (*p* < 0.05) and Biosynthesis of vancomycin group antibiotics (*p* < 0.01) were positively and negatively correlated with *Clostridium_T*, respectively, and these two pathways were negatively correlated with each other (*p* < 0.01). Weaning survival rate was significantly negatively correlated with fecal calprotectin concentration (*p* < 0.01) and farrowing interval (*p* < 0.05). These results describe statistical associations among the measured variables.

## 4. Discussion

In the present study, dietary vitamin D_3_ supplementation during late gestation was used as a short-term nutritional intervention to evaluate its potential effects on maternal intestinal status and subsequent lactation-related outcomes. The main findings were that vitamin D_3_ supplementation improved constipation-related indicators, as reflected by increased fecal scores, higher fecal moisture content, and reduced prepartum and postpartum constipation rates. In addition, fecal calprotectin concentration was reduced in the VD_3_ group; whereas, serum TNF-α was not significantly affected. Although most reproductive and lactation performance indicators were unchanged, piglet weaning survival rate was numerically increased in the VD_3_ group. In modern hyperprolific sows, combined pressures from genetic selection, environmental conditions, and management practices may increase susceptibility to periparturient dysgalactia syndrome [38,39]. Therefore, although the difference in weaning survival rate did not reach statistical significance in the present study, this numerical increase may have biological relevance and warrants further validation in studies with larger sample sizes. Furthermore, exploratory microbiota analysis suggested modest changes in fecal microbial community structure and several genus-level differential taxa. These findings suggest that maternal vitamin D_3_ supplementation may be associated with improved intestinal status in sows during late gestation, but the underlying mechanisms require further investigation.

In the present study, vitamin D_3_ supplementation was applied as a short-term nutritional intervention during late gestation, with the final dietary vitamin D_3_ level reaching 2340 IU/kg. Given the fat-soluble nature of vitamin D_3_ and the potential risk of toxicity associated with excessive intake, a short-term supplementation strategy was adopted rather than prolonged high-level supplementation. The selected dose was based on practical sow feeding conditions and previous studies using comparable or higher dietary vitamin D_3_ levels [40,41]. The intervention window from G 75 to G 90 occurred before farrowing and coincided with a period of maternal physiological adaptation. Previous research has indicated that, from approximately day 69 of gestation onward, fetal growth and development increasingly contribute to maternal phosphorus requirements, accompanied by increased maternal calcium and phosphorus demands [42]. Therefore, this stage may represent a relevant period for evaluating maternal responses to vitamin D_3_ supplementation. Changes in maternal intestinal status during this period may also be relevant to sow health and subsequent lactation-related outcomes [43]. Thus, the observed changes in fecal moisture content, constipation-related parameters, and fecal calprotectin may reflect maternal intestinal responses associated with vitamin D_3_ supplementation before farrowing.

Previous studies on vitamin D nutrition in sows have mainly focused on maternal vitamin D status, reproductive and lactation performance, and offspring growth, immunity, and skeletal development. For example, maternal VD_3_ supplementation during gestation has been reported to improve intestinal development, barrier-related gene expression, short-chain fatty acid production, and microbial composition in weaning piglets [44]. In addition, 25-hydroxycholecalciferol supplementation during late gestation or under different rearing conditions has been shown to affect sow vitamin D status, farrowing performance, antioxidant capacity, immunoglobulin levels, and offspring-related outcomes [45,46]. However, the responses to maternal vitamin D supplementation are not always consistent and may depend on vitamin D source, dose, supplementation period, and physiological status [27]. In line with these reports, most reproductive and lactation performance indicators were not significantly altered in the present study, although the weaning survival rate of piglets from VD_3_-supplemented sows showed a numerical increase. Unlike previous studies that primarily emphasized performance traits or vitamin D status, the present study further focused on maternal intestinal status during the periparturient period. This provides a basis for considering whether changes in maternal intestinal status may be linked to lactation-related offspring outcomes.

The numerical increase in weaning survival rate observed in the VD_3_ group may be partly related to improved maternal intestinal status during the periparturient period. In the present study, VD_3_ supplementation increased sow fecal scores and fecal moisture content and reduced both prepartum and postpartum constipation rates, indicating improvements in fecal characteristics and constipation-related indicators. In addition, fecal calprotectin concentration was significantly reduced in the VD_3_ group; whereas, serum TNF-α concentration was not significantly changed, suggesting that VD_3_ supplementation was more closely associated with local intestinal inflammation-related changes than with systemic inflammatory status. Fecal calprotectin is mainly derived from neutrophils and is widely used as a non-invasive marker of intestinal inflammation [47,48]. Vitamin D_3_ may contribute to intestinal homeostasis through vitamin D receptor-mediated regulation of epithelial barrier integrity, mucosal immune responses, and host–microbiota interactions [49]. Therefore, the reduction in fecal calprotectin may reflect a lower intestinal inflammatory burden or improved mucosal homeostasis in VD_3_-supplemented sows [50]. Correlation analysis further showed that weaning survival rate was negatively correlated with fecal calprotectin concentration and farrowing interval, while fecal moisture content was negatively correlated with constipation rate. Together, these findings support a potential association pathway in which vitamin D_3_ supplementation during late gestation may improve maternal intestinal status, reduce constipation-related stress and intestinal inflammation, and thereby contribute to a more favorable periparturient condition that may support piglet survival during lactation. However, the specific mechanisms underlying this pathway remain to be further elucidated, as intestinal barrier function, neutrophil infiltration, cytokine profiles, short-chain fatty acids, and vitamin D receptor signaling were not directly measured in this study.

Changes in fecal microbiota may represent another component of the maternal intestinal response to vitamin D_3_ supplementation. In this study, alpha diversity indices were not markedly altered, indicating that vitamin D_3_ supplementation did not substantially change overall microbial richness or evenness. Furthermore, beta diversity analysis based on Bray–Curtis distance revealed no significant separation in microbial community structure between the CON and VD_3_ groups, suggesting that vitamin D_3_ supplementation did not induce broad alterations in the overall fecal microbial composition. Nevertheless, vitamin D_3_ supplementation may still influence specific bacterial taxa or microbial functions that are not reflected by global diversity metrics. This interpretation is supported by previous evidence that vitamin D and vitamin D receptor signaling contribute to intestinal barrier integrity, antimicrobial peptide expression, mucosal immune regulation, and host–microbiota interactions [49], suggesting that vitamin D may modulate intestinal homeostasis without necessarily causing large-scale shifts in overall microbial diversity. At the genus level, LEfSe analysis identified several differential taxa, including *Clostridium_P*, *Clostridium_T*, and *Onthenecus*. *Clostridium*-related lineages have been reported in previous pig gut microbiota studies, with *Clostridium_P* detected in piglet fecal or intestinal microbiota and *Clostridium_T* identified as a differential or dominant genus in pig gut microbiota studies [51,52,53]. In sow-related research, *Clostridium_T* was also reported to be associated with oxidative status and reproductive performance indicators; although, these relationships were correlative rather than causal [54]. In the present study, *Clostridium_P* was negatively correlated with constipation rate; whereas, *Clostridium_T* was associated with predicted microbial metabolic pathways. These findings suggest that Clostridium-related lineages may be involved in the microbial changes associated with VD_3_ supplementation and fecal traits. Similarly, *Onthenecus* is a poorly characterized candidate/database-defined genus originally described from metagenomic studies of the chicken gut microbiome [55], and its biological significance in sows remains unclear. Therefore, these taxa may be interpreted as exploratory microbial features associated with VD_3_ supplementation rather than confirmed functional drivers.

The differences in fecal microbial composition were accompanied by changes in predicted microbial functional profiles. Among the predicted KEGG pathways, ansamycin biosynthesis and vancomycin group antibiotic biosynthesis showed nominal differences between the CON and VD_3_ groups. Ansamycins are a class of microbial polyketide natural products that include compounds such as rifamycin, ansamitocin, and geldanamycin; whereas, vancomycin belongs to the glycopeptide antibiotics and is produced through complex microbial biosynthetic pathways [56,57]. Therefore, these pathways may broadly reflect the predicted secondary metabolic potential of the fecal microbiota, rather than indicating the actual production of these antibiotic compounds. This interpretation is consistent with the concept that microbial secondary metabolites can participate in microbial ecological interactions and community adaptation [58,59,60]. In the present study, the nominal differences in these pathways may indicate that vitamin D_3_ supplementation was associated with shifts in the predicted functional potential of the fecal microbiota, possibly as part of a broader maternal intestinal response. This possibility is biologically plausible, as vitamin D has been linked to intestinal barrier function, mucosal immune regulation, and host–microbiota interactions [49]. However, because these pathways were inferred from 16S rRNA gene sequencing data using PICRUSt2, they represent predicted functional potential rather than directly measured metabolic activity [61]. In addition, 16S rRNA-based functional inference has inherent limitations and should not be considered a substitute for shotgun metagenomic or metabolomic analyses [62]. Further studies integrating shotgun metagenomics and metabolomics are therefore needed to determine whether these predicted functional differences correspond to actual microbial metabolic alterations.

## 5. Conclusions

In conclusion, dietary vitamin D_3_ supplementation during late gestation may support intestinal status in periparturient sows, as reflected by reduced constipation-related indicators, lower fecal calprotectin concentration, and changes in fecal microbial composition and predicted functional profiles. Although piglet weaning survival only tended to increase in the VD_3_ group, these findings suggest that maternal vitamin D_3_ nutrition may have potential relevance to lactation-related offspring outcomes. Further studies with larger sample sizes are needed to confirm this potential association.

## Figures and Tables

**Table 1:** Effects of vitamin D_3_ on body weight, backfat thickness and lactation feed intake of sows ^1^.

Item	CON	VD_3_	*p*-Value
No. of sows	20	20	
Parity ^2^	3.05 ± 0.17	2.95 ± 0.15	0.698
Sow backfat thickness, mm			
G 75	15.20 ± 0.47	15.25 ± 0.41	0.936
G 110	15.65 ± 0.40	15.85 ± 0.36	0.711
Farrowing	14.30 ± 0.36	14.55 ± 0.43	0.660
Weaning ^2^	12.70 ± 0.49	13.40 ± 0.49	0.301
Deposition from G 75 to G 110 ^2^	0.45 ± 0.26	0.60 ± 0.28	0.512
Loss during lactation	1.60 ± 0.31	1.15 ± 0.34	0.337
Body weight of sows, kg			
G 75	210.62 ± 5.14	208.74 ± 3.73	0.769
G 110	224.46 ± 3.47	226.03 ± 3.51	0.753
Farrowing	201.43 ± 4.07	200.81 ± 4.28	0.917
Weaning ^2^	179.61 ± 4.19	180.93 ± 3.87	0.904
Deposition from G 75 to G 110	13.84 ± 2.98	17.29 ± 2.95	0.416
Loss during lactation	21.82 ± 2.15	19.88 ± 3.10	0.610
ADFI, kg/d			
Week 1 of lactation ^2^	5.12 ± 0.14	5.36 ± 0.17	0.301
Week 2 of lactation ^2^	6.56 ± 0.24	7.09 ± 0.28	0.211
Week 3 of lactation	7.21 ± 0.19	7.69 ± 0.27	0.154
Lactation	6.30 ± 0.17	6.72 ± 0.21	0.132

^1^ CON = Control group; VD_3_ = Vitamin D_3_ supplementation group, with a dietary vitamin D_3_ activity of 2340 IU/kg from G 75 to G 90. Data are expressed as mean ± standard error of the mean (SEM). Independent samples *t*-test was used for those not stated. ^2^ Data were analyzed by Mann–Whitney U test. ADFI = Average daily feed intake; G 75 = Gestational day 75; G 110 = Gestational day 110.

**Table 2:** Effects of vitamin D_3_ on farrowing performance of sows ^1^.

Item	CON	VD_3_	*p*-Value
No. of litters	20	20	
Parity	3.05 ± 0.17	2.95 ± 0.15	0.698
Birth weight of litter, kg	18.75 ± 0.92	19.03 ± 0.60	0.779
Birth weight of piglet, kg	1.24 ± 0.03	1.27 ± 0.05	0.904
Body weight distribution, kg			
≤0.8	1.90 ± 0.40	1.90 ± 0.35	0.862
0.8–1.2	3.45 ± 0.39	3.75 ± 0.64	0.799
≥1.2 ^2^	9.70 ± 0.60	9.55 ± 0.68	0.869
Total born number ^2^	15.90 ± 0.47	15.75 ± 0.65	0.852
Live-born number ^2^	15.05 ± 0.55	15.20 ± 0.60	0.854
Stillbirths	0.30 ± 0.13	0.40 ± 0.18	0.947
Mummified fetus	0.55 ± 0.20	0.15 ± 0.08	0.231
Live-born piglet rate, % ^3^	94.65	96.51	0.257
Stillborn rate, % ^3^	1.89	2.54	0.577
Between-litter CV, %	21.99 ± 1.67	22.89 ± 1.04	0.738
IUGR piglets, ≤0.92 kg ^4^	1.50 ± 0.14	0.75 ± 0.10	0.758
Farrowing duration, min	319.85 ± 40.37	282.90 ± 19.52	0.678
Inter-piglet farrowing interval, min	20.40 ± 2.51	18.29 ± 1.28	0.659

^1^ CON = Control group; VD_3_ = Vitamin D_3_ supplementation group, with a dietary vitamin D_3_ activity of 2340 IU/kg from G 75 to G 90. Data are expressed as mean ± standard error of the mean (SEM). Mann–Whitney U test was used for those not stated. The sow/litter was used as the experimental unit for reproductive and piglet-related outcomes. Thus, 20 sows/litters per group were included in the final analysis, and piglet-related variables are presented at the litter level. ^2^ Data were analyzed by independent samples *t*-test. ^3^ Data were analyzed by Chi-square test. ^4^ IUGR is defined in piglets by the diagnostic criterion that their birth weight is two standard deviations below the mean value (mean − 2SD). In this study, the birth weight of the 40 litters of piglets was 1.26 ± 0.17 kg. IUGR piglets were defined as those with a birth weight ≤ 0.92 kg. IUGR = intrauterine growth restriction; CV = coefficient of variation.

**Table 3:** Effects of vitamin D_3_ on growth performance of piglets ^1^.

Item	CON	VD_3_	*p*-Value
No. of litters	20	20	
No. of piglets			
Within 48 h	12.05 ± 0.34	11.95 ± 0.28	0.819
Day 21 of lactation ^2^	11.30 ± 0.37	11.60 ± 0.23	0.314
Average weight of piglets, kg			
Within 48 h	1.34 ± 0.04	1.36 ± 0.04	0.758
Day 21 of lactation	5.94 ± 0.15	5.99 ± 0.17	0.824
ADG, g/d	219.23 ± 6.64	220.82 ± 7.32	0.874
Litter weight, kg			
Within 48 h	16.12 ± 0.69	16.21 ± 0.61	0.930
Day 21 of lactation	67.38 ± 3.04	69.39 ± 2.24	0.597
Gain	51.26 ± 2.49	53.19 ± 1.84	0.537
Weaning survival rate ^3^	93.78	97.07	0.084

^1^ CON = Control group; VD_3_ = Vitamin D_3_ supplementation group, with a dietary vitamin D_3_ activity of 2340 IU/kg from G 75 to G 90. Data are expressed as mean ± standard error of the mean (SEM). Independent samples *t*-test was used for those not stated. ^2^ Data were analyzed by Mann–Whitney U test. ^3^ Data were analyzed by Chi-square test. ADG = average daily gain.

## Data Availability

The datasets used during the current study are available from the corresponding author on reasonable request.

## Supplementary Materials

The following supporting information can be downloaded at: https://www.mdpi.com/article/10.3390/ani16172690/s1, Table S1: The composition and nutrient levels of the diets.
