# Supplementary material for: The Effects of Vitamin D3 Supplementation in Late Gestation on Lactation Performance, Constipation and Intestinal Microbiota of Sows
Source: Animals (Basel). 2026 Aug 28;16(17):2690. doi: 10.3390/ani16172690 (PMC13564314; doi:10.3390/ani16172690)
Supplement: Supplementary file 1 [file animals-16-02690-s001.zip › animals-4427396-supplementary.pdf]

Supplementary Table S1

Diet composition of sows.

Item	Unit	Gestation diet[a]	Lactation diet[b]
Ingredient, %			
Barley	%	31.99	-
Cassava	%	20.00	5.00
Bran	%	19.33	-
Palm meal	%	8.00	-
Sorghum	%	7.72	5.00
Soybean meal (43 %)	%	5.80	24.26
Distillers' grains	%	2.00	-
Limestone powder	%	1.55	1.59
Corn		-	58.47
Soybean oil		-	1.31
Rice bran meal		-	1.29
Lys (98 %)	%	0.31	0.33
Met	%	0.09	0.12
Thr	%	0.13	0.12
Trp		-	0.03
Val		-	0.01
Premix	%	3.08	2.47
Measured composition, %			
CP	%	12.70	16.18
EE	%	2.29	3.71
Crude ash	%	6.07	5.39
CF	%	6.28	3.09
NE	kcal/kg	2024.30	2468.70

DM = dry matter; CP = crude protein (N × 6.25); EE = ether extract; CF = crude fiber; Ca = calcium; AP = available phosphorus; NE = net energy.

[a] Per kilogram of feed provides: vitamin A, 6000 IU; vitamin D_3, 1000 IU; vitamin E, 60 IU; vitamin K_3, 2.0 mg; vitamin B_1, 1 mg; vitamin B_2, 7 mg; vitamin B_6, 5 mg; vitamin B_{12}, 0.02 mg; niacinamide, 30 mg; pantothenic acid calcium, 20 mg; folic acid, 4 mg; biotin, 0.5 mg.

[b] Per kilogram of feed provides: vitamin A, 6000 IU; vitamin D_3, 1000 IU; vitamin E, 60 IU; vitamin K_3, 2.0 mg; vitamin B_1, 1 mg; vitamin B_2, 7 mg; vitamin B_6, 5 mg; vitamin B_{12}, 0.02 mg; niacinamide, 30 mg; pantothenic acid calcium, 20 mg; folic acid, 4 mg; biotin, 0.5 mg.
